# An Allosteric Cross-Talk Between the Activation Loop and the ATP Binding Site Regulates the Activation of Src Kinase

**DOI:** 10.1038/srep24235

**Published:** 2016-04-11

**Authors:** Encarna Pucheta-Martínez, Giorgio Saladino, Maria Agnese Morando, Jorge Martinez-Torrecuadrada, Moreno Lelli, Ludovico Sutto, Nicola D’Amelio, Francesco Luigi Gervasio

**Affiliations:** 1 Department of Chemistry, University College London, London WC1E 6BT, United Kingdom; 2 Research Institute of Structural and Molecular Biology, University College London, London WC1E 6BT, United Kingdom; 3 Center of Technological Development in Health, Oswaldo Cruz Foundation (Fiocruz), Rio de Janeiro, Brazil; 4 Structural Biology and Biocomputing Programme, Spanish National Cancer Research Centre (CNIO), 28029, Madrid, Spain; 5 Centre de RMN à Très Hauts Champs, Institut de Sciences Analytiques, (CNRS/ENS Lyon/Universitè CB Lyon 1), 69100 Villeurbanne, France

## Abstract

Phosphorylation of the activation loop is a fundamental step in the activation of most protein kinases. In the case of the Src tyrosine kinase, a prototypical kinase due to its role in cancer and its historic importance, phosphorylation of tyrosine 416 in the activation loop is known to rigidify the structure and contribute to the switch from the inactive to a fully active form. However, whether or not phosphorylation is able per-se to induce a fully active conformation, that efficiently binds ATP and phosphorylates the substrate, is less clear. Here we employ a combination of solution NMR and enhanced-sampling molecular dynamics simulations to fully map the effects of phosphorylation and ATP/ADP cofactor loading on the conformational landscape of Src tyrosine kinase. We find that both phosphorylation and cofactor binding are needed to induce a fully active conformation. What is more, we find a complex interplay between the A-loop and the hinge motion where the phosphorylation of the activation-loop has a significant allosteric effect on the dynamics of the C-lobe.

Protein kinases (PK) are a large and diverse family of enzymes that catalyze the transfer of the γ-phosphate group from ATP to specific tyrosine, serine or threonine residues^1^,^2^. Due to their central role in regulating essential cellular processes^3^, the catalytic activity of PKs is strictly regulated under physiological conditions^4^,^5^. In response to external signals, PKs alternate between a catalytically active conformation and one or more inactive conformations. When the controlled switching mechanism is altered by pathogenic mutations, it may result in unregulated cellular growth and proliferation. Indeed PKs are one of the most commonly mutated protein families in cancer^6^, explaining their importance in anti-cancer drug discovery.

The catalytic domain of PKs assumes a common fold consisting of two lobes, a smaller N-terminal lobe and a larger C-terminal one^7^,^8^. Two conserved hydrophobic structures termed “spines” (the regulatory or “R” spine and the catalytic or “C” spine) traverse the two lobes of PKs and dynamically anchors all the elements important for catalysis to the central F-helix^9^. The transition from the inactive to the active form involves complex conformational changes in at least three conserved structural motifs at the active site: the activation loop (A-loop), the Asp-Phe-Gly (DFG) motif and the αC-helix (Fig. 1)^5^,^10^. The conformational changes contribute to the full assembly of the two hydrophobic spines and make the ATP binding cleft at the interface between the two lobes accessible to the substrates^11^,^12^,^13^. The process is initiated and controlled by different mechanisms, one of the most common being the phosphorylation of the A-loop. The A-loop is located in the C-terminal part of an extended activation segment, which is the most critical important regulatory element in PKs. In most non-constitutively active PKs the A-loop contains a phosphorylation sites (the primary phosphorylation site). From a structural point of view, the phosphate makes a set of strong hydrogen bonds and electrostatic contacts linking the A-loop with both the catalytic site and the substrate-binding surface^1^,^7^,^10^,^14^. The structural re-organization improves the binding of the substrate peptides^11^,^15^,^16^,^17^,^18^.

At the same time, H/D exchange experiments on PKA^18^ and molecular dynamics simulations on Src^14^ have shown a profound effect of phosphorylation on the flexibility of the structure, which turns out to be more rigid, particularly at the A-loop. The molecular mechanism emerging from these studies is that the un-phosphorylated A-loop is highly flexible^7^,^8^,^11^,^15^. After phosphorylation, it is locked in a catalytically competent conformation. It might thus seem that the rigidification of the A-loop is both necessary and sufficient for the phosphorylation-dependent activation.

However, it has been long known that both inter-lobe hinge dynamics (clamshell-like)^9^,^19^,^20^,^21^ and allosteric effects^11^,^12^,^13^ play an important role in regulating the catalytic activity of PKs. In Src an allosteric network connecting the ATP and substrate binding sites has been observed^22^, while in homologous TK a cross-talk between the SH2 and SH3 regulatory domains and the phosphorylation (Hck) and active site (Abl) has been proposed^17^,^20^,^23^,^24^. Recent NMR studies on MAP kinase have shown an allosteric link between the phosphorylation sites and the hinge motion, whereby both ATP-loading and phosphorylation are necessary to induce a fully active conformation^10^. Albeit a close connection between the A-loop switch and the hinge has also been shown in the EGFR kinase^25^, it is still not clear whether these allosteric effects are general and what is their molecular mechanism. To address these questions, here we combine solution NMR and enhanced-sampling molecular simulations to study the conformational dynamics of Src kinase phosphorylated and un-phosphorylated with and without a bound cofactor.

The Src cytoplasmic non-receptor protein kinase, the first viral oncogenic protein discovered, is an ideal system for mechanistic explorations of PKs due to its high conformational flexibility, biomedical and historical importance^3^,^24^ as well as the availability of extensive experimental and computational data^4^,^5^,^14^,^26^, including crystal structures in both the inactive (PDB id: 2SRC)^15^ and active conformations (PDB id: 1Y57, 1YI6)^27^. Src regulates cell growth, proliferation and migration, it is involved in metastasis and is mutated in 50% of colon, liver, lung, breast and pancreas tumors. Thus, a better understanding of its activation mechanism by tyrosine 416 phosphorylation^9^,^28^ and substrate binding not only will help addressing the open questions on the allosteric cross-talk in PKs, but could also help the design of more effective Src inhibitors.

## Results and Discussion

The NMR assignment of Src catalytic domain (Table S1; BMRB entry 25756) reveals that some parts of the protein are not detectable (the ^1^H,^15^N TROSY NMR spectrum of the triply isotopically labelled sample at 1 GHz is shown in SI Fig. S1, superimposed to the spectrum of its phosphorylated form). Out of the 268 backbone amides only about 200 are visible in the spectrum of the dephosphorylated Src catalytic domain. Most of the missing peaks (42 out of 68) belong to the functionally relevant A-loop (residues 403–429) and the αC helix (residues 303–317). As exchange with the solvent can be mostly ruled out at a pH of 6.5, disappearance of the signals beyond detection can be attributed to significant conformational exchange in the μs-ms time-scale. Thus, the A-loop and the αC helix experience a significant conformational dynamics, in agreement with the previously reported structural heterogeneity of these regions^29^.

A close look to the TROSY spectrum of Src at 1 GHz reveals the presence of a second species in slow conformational exchange (Fig. 2). The doubling of peaks, which has been observed thanks to the high magnetic field NMR data, is apparent for selected residues of the protein. The location of the affected peaks, mainly at the inter-lobe interface (green residues in Fig. 2), seems to suggest that the two forms might be related to the hinge motion and is consistent with our computational results (see further in the text).

Phosphorylation of Src can be readily followed by NMR monitoring the changes in the ^1^H-^15^N TROSY spectrum upon addition of a solution of ATP to a sample of ^15^N labelled Src. The reaction can be followed using G437 and G530 as isolated probes. The signal of G437 resides in a non-crowded region of the spectrum and its location in the structure, just below the phosphorylation site, makes it a good probe to check the phosphorylation state of the protein (see SI Fig. S3). A further phosphorylation site, in position 527 (which can be monitored by the close G530) it is not affected by the addition of ATP as no shift in that region is observed (see SI Fig. S3), in agreement with MS results indicating phosphorylation only on residue 416 (see SI Fig. S4). After the full phosphorylation is achieved, the cofactor is washed away to obtain the spectra of the phosphorylated apo-form (SrcP). The differences are evident (see SI Fig. S1) as at least 30 new main chain peaks appear in the spectrum. These signals might arise from the activation loop or the αC helix. Indeed, a reduction in conformational disorder in the activation loop upon phosphorylation, has been previously suggested by H/D exchange experiments in PKA^18^, computational predictions^14^, and crystallography^9^. The low intensity, however, prevents us from unequivocally assign the new resonances, due to the loss of inter-residue information in triple resonance spectra. Nonetheless, a tentative assignment of the full activation loop and part of the αC helix (see Table S2) is obtained once a few assumptions are made (see also the experimental section in SI). The first is that the new resonances probably arise from regions that were disordered in the unphosphorylatyed protein and the second is that the structure of the activation loop is similar to the one assumed in the crystal structure and simulations. The latter assumption allowed us to use theoretical prediction of chemical shifts to suggest the most likely candidate for a particular residue (see also SI). In this way we could tentatively assign almost the full stretch of missing resonances belonging to the activation loop, in agreement with its structuring upon phosphorylation. In some cases, the assignment is further supported by inter-residues connectivities in the spectra. A further indication that the new signals may indeed belong to the activation loop is given by glycine resonances. Out of the three unassigned glycine residues, one is at the N terminus (and it is not observable), while the others two belong to the activation loop and are consistent with glycine-like spin systems as reported in Table S2.

**Figure 1:**
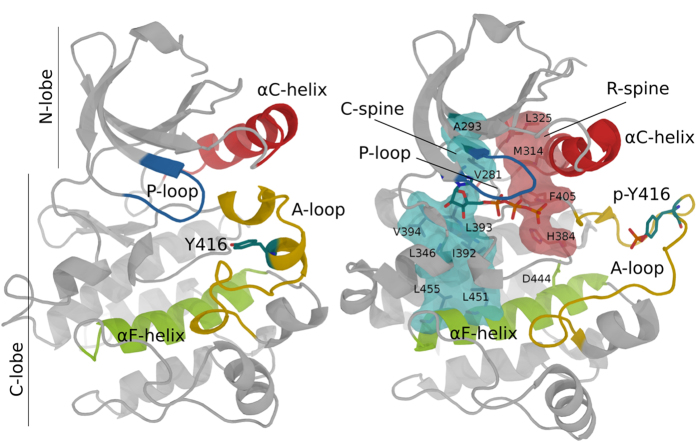
Comparison of inactive (Left, PDB id: 2SRC) and phosphorylated, active (Right, PDB id: 1YI6) crystal structures of the Src catalytic domain. Key structural elements are colored in red (αC helix), blue (P-loop), yellow (A-loop) and green (αF helix). The (P)-Tyr416 and ATP are shown as sticks. The C- and R-spines are shown as surfaces (with inner residues as sticks) in cyan and pink, respectively.

**Figure 2:**
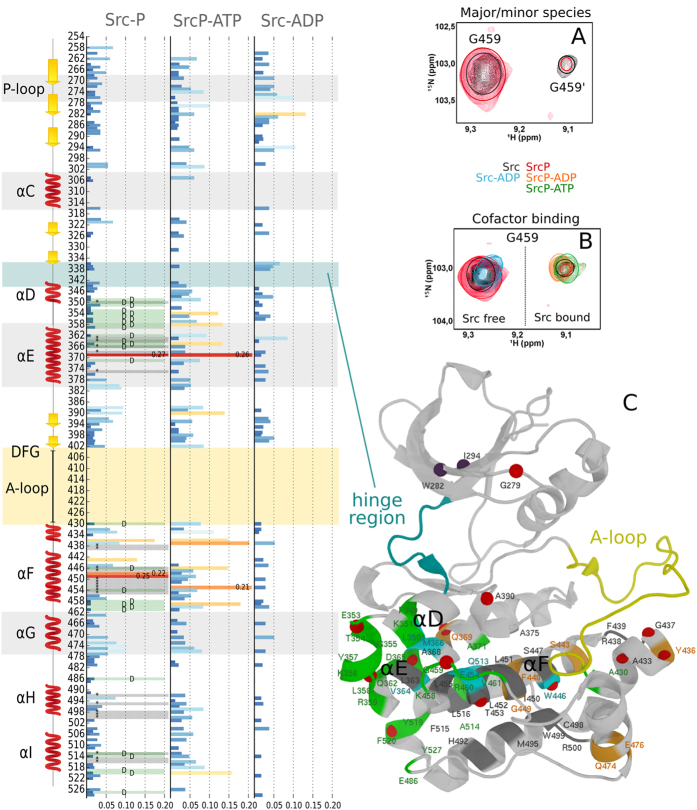
(left panel) Combined chemical shift deviations of SrcP, SrcP-ATP and Src-ADP complexes with respect to free Src. Doubling of peaks is indicated with a letter D and a green bar, while peaks disappearing are reported with a * sign and a grey bar. (right panel) (**A**) Src (black) and SrcP (red) exists in two unevenly populated forms in solution as exemplified by G459 (but also by many other peaks highlighted in green in the structure) in the ^1^H,^15^N TROSY NMR spectrum; (**B**) the minor form (indicated by G459′ in A) becomes prevalent with the addition of the cofactor (ATP/ADP, green and orange) only if the protein is phosphorylated. Addition of ADP to free Src (light blue) forms a weaker complex causing only a small shift, even in excess of the ligand; (**C**) the crystal structure of Src (1YI6) showing the residues for which the doubling of peaks is apparent in green, peaks disappearing upon phosphorylation in grey and peaks that have a high deviation in SrcP in orange. Peaks with high deviation in SrcP-ATP and Src-ADP are also shown as red and purple spheres, respectively. Coloured circles in spectra have been added to better clarify the position of the peaks.

The equilibrium between the major and the minor conformations observed in unphosphorylated Src is still present, as shown by the doubling of some of the SrcP peaks (Fig. 2A). However, the disappearance of some peaks (see Fig. 2 and SI Fig. S2) seems to suggest that the inter-conversion rate changes upon phosphorylation. Phosphorylation also causes substantial chemical-shift perturbations (CSPs) and severe line broadening to a number of other resonances, mainly from residues located in the C lobe and at the interface between the lobes (see Fig. 2 and SI Fig. S2). Interestingly, significant CSPs are observed for the N-ter regions of the αE and αF, corresponding to the location of the C-spine (see Fig. 1), whose residues V281 and L393 are close to the ATP binding site and, indeed, change further in the presence of ATP or ADP (see Fig. 2).

Of the assigned residues of the R-spine, whose correct assembly is necessary for the catalytic activity, only L325 changes. This residue is close to the αC helix and may reflect a change in its conformation. Indeed, residues 300 and 301 at the beginning of the helix display changes in shift and intensity.

Overall, global changes in the spectra are in agreement with a conformational change in the A-loop and a rigidification of the region, together with a change in the inter-conversion rate between the major and minor conformers. Interestingly, the signals from the P-loop and from K295, which makes a salt bridge to E310 in the active form but not in the inactive form, remain unaltered (instead, these regions are affected by the presence of ADP or ATP, see SI Fig. S5). This might suggest that, as previously observed in the MAP kinase^10^, the full active conformation is not achieved in the absence of the ATP cofactor.

**Figure 3:**
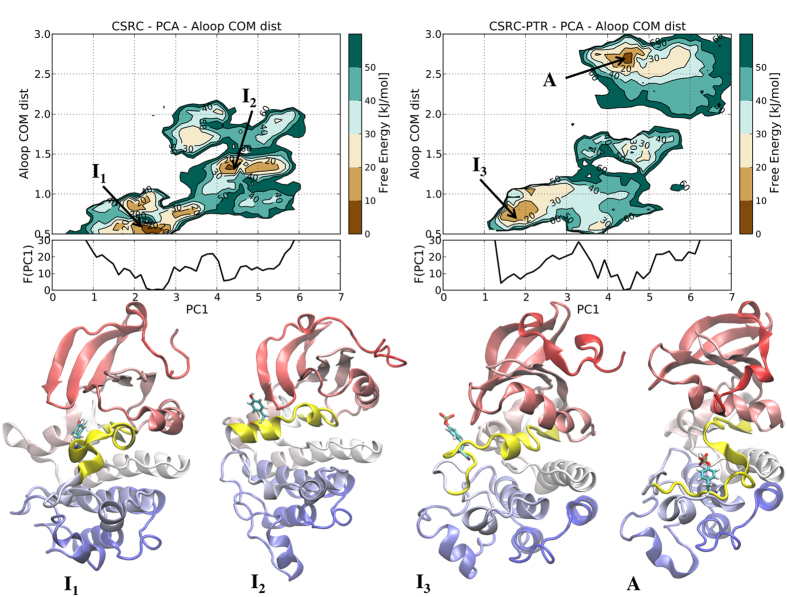
Free Energy Surfaces (FESs) of unphosphorylated Src (left) and phosphorylated Src (right) as a function of the first principal component vector (PC1) and the distance of the center of mass of A-loop residues 411–413 to residues 301–303. Below the bi-dimensional plots is shown the mono-dimensional free energy profile along PC1. The central cluster conformations of the main FES minima (the inactive-like, I1, I2 and I3 and active-like, A, structures respectively) are also reported with the A-loop colored in yellow and the Tyr416 (or P-Tyr416 in case of phosphorylated Src) shown with sticks.

To provide a more detailed mechanistic insight on the effects of phosphorylation and confirm the interpretation of the NMR observations, we performed long atomistic MD simulations and free energy calculations. A comparison of the root mean square fluctuations (RMSF) of the phosphorylated and unphosphorylated forms of Src (SI Fig. S6), shows a slight local rigidification of the A-loop (and a much more significant one of the αG-helix), in agreement with the NMR spectra and previous reports^14^,^18^,^29^. In order to assess the effect of the phosphorylation on the main collective motions of the catalytic domain, we performed a principal component analysis (PCA) on the trajectories of the two proteins. The first collective motion, along the PC1 vector, is a hinge motion of the two lobes while the second main motion is a twist of the N-lobe with respect to the C-lobe, as usually observed in TK^20^. The main hinge motion is observed in both the unphosphorylated and phosphorylated Src in agreement with the NMR findings. Furthermore, we see that phosphorylation of Tyr 416 enhances the collective hinge motion while slightly affecting the equilibrium towards a less compact N-lobe/C-lobe positioning (see below). As standard atomistic molecular dynamics (MD) simulations lasting a few hundreds of ns are not able to adequately sample the large conformational changes associated with the inactive-active switch, we performed enhanced-sampling MD simulations (with a combination of parallel-tempering and metadynamics or PT-metaD) for both SrcP and Src catalytic domain (see full Materials and Methods in the SI). To correlate the effects of phosphorylation to both the dynamics and the structure of the catalytic domain and in particular of the A-loop, we calculated the bi-dimensional free energy surface (FES) as a function of the hinge motion (PC1) and the distance between A-loop residues 411–413 and residues 301–303 (see Fig. 3). The latter was identified as the largest varying distance in the inactive-to-active transition.

As shown in Fig. 3, phosphorylation stabilizes the fully active, open and extended, A-loop structure (main minimum A in Fig. 3) even though the closed, inactive-like, conformation is still present albeit to a reduced extent (I3). While the inactive A-loop closed conformation, similar to the crystal structure (Pdb id: 2SRC), is the most stable in the case of Src, it is interesting to note that a secondary minimum (I2 in the figure) is also populated where the residues 413 to 420 of the A-loop form a stable α-helix. This intermediate conformation is never observed in the phosphorylated SrcP. It is also interesting to note that the two minima in each FES correspond to open and closed structures around the hinge, as shown by the different values of PC1. The energy difference between the two states in both Src and SrcP is small. This suggests that the two forms observed for many residues nearby the hinge region (e.g. G459, see Fig. 2 and SI Figs S2 and S3) in the spectra of Src and SrcP is a result of the hinge motion. Moreover, the significant free energy barrier between the two states (which is even higher in SrcP) is in agreement with a slow transition, explaining the disappearance of many NMR peaks at the interface between the lobes. Overall, our results strongly suggest that, while a shift towards the A-loop open structure is observed upon phosphorylation, the equilibrium between hinge-open and closed structures is still present.

### Phosphorylation state and the role of cofactors

To understand the role of the cofactor in achieving the fully active conformation, we recorded NMR spectra of Src and SrcP bound to ATP and ADP. Upon addition of ATP to unphosphorylated Src, we observe a fast trans-autophosphorylation of Src. Indeed, phosphorylation is achieved within minutes from the addition, as observed by NMR spectra and confirmed by MS (SI Fig. S4), leading to the spectrum of SrcP-ADP complex.

Addition of an excess of ATP produces the spectrum of phosphorylated Src bound to ATP (SrcP-ATP). When titrating SrcP with ATP, the changes in the ^1^H,^15^N TROSY spectrum are evident (see CSP in Fig. 2 and SI Fig. S2). Among many changing signals, G459, G344 and G279 (Fig. 2B and Fig. S3) provide good isolated probes to monitor the presence of the cofactor in its binding site (see SI Fig. S3). While the position of G459 can be used to indirectly monitor the presence of ATP or ADP in the binding pocket (see Fig. 2B), G344 and G279 allow the distinction between the two (see Fig. S3C) as they disappear in the presence of ATP. This is not surprising, since the glycine rich P-loop (in which G279 is located) is known to position the γ-phosphate of ATP.

Most importantly, the signals of SrcP complexed with ATP appear in very similar positions to the second form observed in both Src and SrcP (see for instance G459 in Fig. 2A,B; a similar behavior is observed for the other doubled peaks, see SI Fig. S7), indicating that the addition of the cofactor makes the minor form prevalent. This is also true for the ADP complex (SrcP-ADP, see orange peak in Fig. 2B) but not for its unphosphorylated form (Src-ADP, see blue peak in Fig. 2B), demonstrating that both phosphorylation and cofactor binding are needed for the conformational switch.

Relaxation data of the phosphorylated protein in the presence of ATP (see experimental section in SI and Fig. S9) indicate a smaller rotational correlation time (20 ns) with respect to the one estimated for the free protein (26 ns), further supporting a more compact structure, compatible with the closing of the lobes as seen in our simulations.

It has been reported that, in the absence of phosphorylation, the affinity of Src for ADP is negligible^22^, in agreement with the structure of the main conformer predicted by the free energy calculations that, with a semi-closed A-loop and a partially closed hinge, is not compatible with cofactor binding (Fig. 3). This is nicely reflected in the CSP of the diagnostic peaks: when Src is unphosphorylated, the signal of G459 is only slightly affected, even in large excess of ADP (Fig. 2B, light blue peak) suggesting that binding is negligible. Furthermore, the signal of K295, whose positively charged side chain is known to interact with ADP phosphate, does not disappear from the spectrum as in the case of the SrcP-ATP or SrcP-ADP complexes (see SI Fig. S5).

It has been reported that ATP and ADP have opposite cooperativity effects on substrate binding in the case of unphosphorylated Src^22^. Our data show that binding of ADP depends on the phosphorylation state of the protein and that the addition of the ATP/ADP cofactor to SrcP induces a switch to the conformation of the minor form already present in solution. An interpretation of the nature of the minor form is given by the crystal structure of Src in complex with AMP that shows a significant inter-lobe rotation towards a more closed state. Long MD simulations of SrcP in the presence of ATP confirm a closure of the two lobes around the hinge due to the presence of ATP (see SI Fig. S8).

## Conclusions

**Figure 4:**
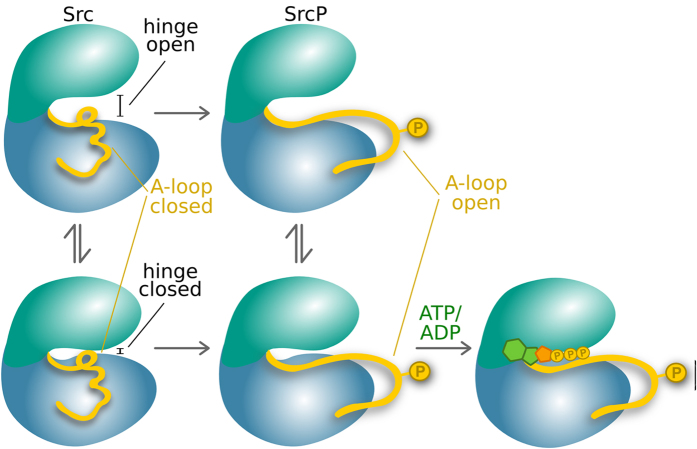
Scheme describing the different conformational states of Src. The free protein is in slow exchange between two conformations: hinge-open (top) and closed (bottom). This equilibrium remains even after the phosphorylation of the activation loop (represented by a yellow sphere). Phosphorylation causes a conformational change leading to the opening of the A-loop. Binding of ATP (or ADP) leads to a final conformational switch towards the minor form observed in solution.

The TK Src exists in solution in at least two different conformations, in slow exchange with one another. Of the two conformers, the less populated one, is remarkably similar to the one induced by binding to ATP, which we now know to be the hinge closed form. Phosphorylation of Y416 on the A-loop has only a slight effect on the hinge closed/open equilibrium, while clearly determining a change in the inter-conversion rate, reflected in both the significant changes of the C-lobe resonances and the barrier in the free energy landscape. At the same time phosphorylation rigidifies the A-loop and stabilizes its extended open conformation, in agreement with previous observations on PKA kinase^14^,^18^ and computer simulations on Src^11^,^14^,^15^. The structural and dynamical changes resulting from the phosphorylation also determine an increase in affinity for ADP. Finally, both the simulations and the experiment show that only the binding of the cofactor (ADP/ATP) shifts the conformational equilibrium of Src towards the fully active form that becomes prevalent in solution. This is consistent with recent reports from NMR solution studies of MAP kinase^4^,^5^,^10^. Overall, the activation model of Src that we propose based on simulations and experiments is as it follows. Initially ATP binds poorly to the kinase. However, once the protein is phosphorylated, not only the kinase is more active (and the A-loop open), but also its affinity towards new ATP molecules is higher, generating a self-enhancing phosphorylation reaction that would trigger signal amplification within the cell. Our model (schematically represented in Fig. 4) reinforces previous reports of a conserved allosteric network connecting the A-loop and the catalytic cleft^22^ and explains the significant enhancement of the catalytic turn-over observed upon phosphorylation.

## Materials and Methods

Samples were prepared in 20 mM phosphate at pH 6.5, 0.5 M NaCl, 1 mM MgCl_2_ , 1 mM TCEP and the sample concentration was 200 μM. NMR experiments were performed on Bruker Avance 500, 700, 800, 950 and 1 GHz, some of them equipped with cryogenically-cooled triple resonance probe. 1H and 15N backbone assignment of the catalytic domain of phosphorylated Src was performed though a series of three-dimensional experiment, namely: trHNCO, trHNCA, trHNCACB and ^1^H,^15^N-NOESY-HSQC recorded on singly or triply labeled samples with deuteration >70%. The assignment was aided by the comparison with the assignment of the unphosphorylated form (BMRB deposition 25756). The molecular dynamics simulations were performed with the GROMACS simulation package^30^. All free energy calculations were performed with PT-metaD^31^ using GROMACS and the PLUMED plugin^32^ as in previous studies of kinase conformational changes^33^,^34^. Full Materials and Methods are detailed in SI.

## Additional Information

**How to cite this article**: Pucheta-Martínez, E. *et al*. An Allosteric Cross-talk Between the Activation Loop and the ATP Binding Site Regulates the Activation of Src Kinase. *Sci. Rep*. **6**, 24235; doi: 10.1038/srep24235 (2016).

## Supplementary Material

Supplementary Information
