# Supplementary material for: An Allosteric Cross-Talk Between the Activation Loop and the ATP Binding Site Regulates the Activation of Src Kinase
Source: Sci Rep. 2016 Apr 11;6:24235. doi: 10.1038/srep24235 (PMC4827121; doi:10.1038/srep24235)
Supplement: Supplementary Information [file srep24235-s1.pdf]

Supplementary Information: An Allosteric Cross-talk Between the Activation Loop and the ATP Binding Site Regulates the Activation of Src Kinase

Encarna Pucheta-Martínez[+], Giorgio Saladino[+], Maria Agnese Morando, Jorge Martinez-Torrecuadrada, Moreno Lelli, Ludovico Sutto, Nicola D'Amelio*, Francesco Luigi Gervasio*

Materials and Methods

Bacterial expression Src-KD

The kinase domain of c-Src (residues Q251–L533)[1] were sub-cloned using NdeI and XhoI restriction sites into a pET-28a vector (Novagen) modified to yield a tobacco etch virus (TEV) protease cleavable N-terminal hexahistidine tag. We co-expressed Src-KD with full length YopH phosphatase from Yersinia[2] in order to produce high amounts of soluble and to maintain the kinases in the dephosphorylated state. The two plasmids were co-transformed into E. coli strain BL21(DE3) cells, that was also transformed with the chaperone plasmid pG-KJE8 (Takara) encoding the groESL proteins. 2H,13C,15N-labelled protein was expressed in 100% D2O M9 minimal media (containing 1 g/L of 15NH4Cl as the sole source of nitrogen and 3 gr/L of labelled glucose, 50 mg/ml kanamicin, 50 mg /ml streptomycin, 20 mg/ml chloramphenicol and 5 µg/ml tetracycline). Cultures were grown to an OD600nm of 0.8 at 37 °C and induced overnight at 20 °C with 0.2 mM IPTG. Cell suspensions were clarified by 15 min centrifugation at 4000g. Frozen pellets at -20ºC were re-suspended in 40 ml of lysis buffer (25 mM TRIS pH 8, 250 mM NaCl, 1mM MgCl2, 0.1 mM CaCl2, 10 mM Benzamidine, 40 mM imidazole, lysozyme and subsequently DNAse I and 10% Triton X-100) per liter of cells for immediate purification by immobilized metal affinity chromatography. Insoluble protein and cell debris was sedimented through a 40 min centrifugation at 40,000g at 4ºC. The supernatant was filtered through a 0.45 mm filter and loaded onto a Ni affinity column (HisTrap FF, GE Healthcare), equilibrated with buffer A (50 mM Tris (pH 8.0), 500 mM NaCl, 5% glicerol (v/v), 25 mM imidazole). The loaded column was washed with five column volumes of buffer A, and protein was eluted with a linear gradient of 0–100% of buffer B (buffer A plus 0.5 M imidazole). The peak fractions were analyzed by SDS-PAGE, and fractions containing the kinase were pooled and cleaved with 1 mg of TEV per 20 mg of crude kinase by overnight incubation at room temperature. The solution was loaded onto a HisTrap column to separate the protein from the cleaved tag. The main impurity at this stage was Yop phosphatase, which tends to bind to the Ni affinity resin despite the lack of a histidine tag. Subsequent anion exchange chromatography was used to remove protease and phosphatase contaminants. The protein was diluted five-fold and loaded onto an anion exchange column (HiTrap Q FF, GE Healthcare), equilibrated with 20 mM Tris (pH 8.0), 5% glycerol, 1 mM DTT (buffer QA). Protein was eluted with a linear gradient of 0–100% buffer QB (buffer QA plus 1 M NaCl). Fractions containing the target protein were pooled again together and loaded onto a size-exclusion column (Superdex 75 16/60, GE Healthcare) equilibrated with 20 mM Tris (pH 8), 0.5 M NaCl, 1 mM MgCl2, and 1 mM TCEP.

Protein was concentrated to 10 mg/mL, frozen in liquid nitrogen, and stored at -80ºC. Concentration of the proteins was determined by absorbance spectroscopy at 280 nm using calculated extinction coefficient of 52,370 M-1 cm-1 for Src. The protocol yields about 12 mg of purified Src- KD and active kinase domain per liter of bacterial culture. The identity of the protein was confirmed by mass spectrometry, and showed no evidence for post-translational modification. Mass spectrometry demonstrated the incorporation of the label and HSQC NMR spectra showed that the kinases were folded (data not shown).

Src-KD phosphorylation

Phosphorylation of the pure protein takes place in a few minutes after addition of a concentrated solution of ATP in equimolar ratio. This was conformed by enzymatic digestion followed by MS and by NMR, once the spectrum of the phosphorylated protein was known.

As we mentioned above Src implies the co-expression of YopH phosphatase. Thus, an accurate and suitable purification of the protein is essential for phosphorylation experiments. This requires an extra step of purification (with respect to the protocol shown above) involving the use of Q column after the addition of ATP. Extra ATP deriving from the eluate can be removed by subsequent cycles of centrifugation with 10-kDa molecular cut-off membrane.

High field NMR

Samples were prepared in 20 mM phosphate at pH (6.5), 0.5 M NaCl, 1 mM MgCl2 , 1 mM TCEP and the sample concentration was 200 μM. The temperature was set to 293 K and the choice of the pH was dictated by a compromise between protein solubility (decreasing at lower pH) and the detectability of water-exchanging amide protons. NMR experiments were performed on Bruker Avance 500, 700, 800, 950 and 1 GHz, some of them equipped with cryogenically-cooled triple resonance probe. ^{1}H and ^{15}N backbone assignment of the catalytic domain of phosphorylated Src was performed though a series of three-dimensional experiment, namely: trHNCO, trHNCA, trHNCACB and $^{1}H,^{15}N$-NOESY-HSQC recorded on singly or triply labeled samples with deuteration > 70%. The assignment was aided by the comparison with the assignment of the unphosphorylated form (BMRB deposition 25756), free or in the presence of Imatinib (Ref. [3]).

Analysis of new peaks arising from phosphorylation

A possible assignment of new spin systems appearing in the spectra upon phophorylation of Src was obtained by making two assumptions. The first is that the new resonances must arise from previously disordered regions in the unphosphorylatyed protein and the second is that the structure of the activation loop is similar to the one described by its crystal structure. The first assumption allowed us to discard missing residues from regions different from the long activation loop and the alpha C-helix. The second allowed us to use theoretical prediction of chemical shift to suggest the most likely candidate for a particular residue. Although we are aware of the questionability of such assumptions, we found in this way almost the full stretch of missing resonances belonging to the activation loop, indicating that out data are compatible with its structuring upon phosphorylation.

Theoretical values of chemical shifts based on the structure of the phosphorylated protein (pdb code 1YI6, considering the phosphotyrosine as a tyrosine), were obtained by SPARTA+ program [13]. Each spin system (comprising HN,N,C_α,C_β,$C_{\alpha-1}$,$C_{\beta-1}$ and C'_{-1} frequencies when detectable) was previously examined according to the compatibility of its frequencies with the aminoacid sequence, using a tolerance of ± 3.5 ppm in carbon from the average values of the BMRB database.

The overall agreement of each spin system with residue prediction Δδ was calculated as [14]:

$$\Delta\delta_{BA} = \frac{1}{N}\sqrt[2]{\sum_{i=1}^{N}[w_i\,(\delta_{Bi} - \delta_{Ai})^2]}$$

where N is the number of resonances found for each spin system i (including the frequencies of the previous residue), w is the weighting factor depending on the nucleus examined, δ is the chemical shift and A, B refer to the experimental (B) and theoretical value (A). The same equation was used for the calculation of the combined amide chemical shift differences between different states of Src. This overall score allowed sorting candidate residues as reported in Table S2.

Estimation of the rotational correlation time

A way to probe the shape of a protein in solution is to estimate the overall correlation time of its tumbling. Structures which are more compact tend to encounter less friction and tumble faster. For example, the estimated rotational tumbling obtained by the crystal structure of the open form (pdb 2SRC) and the closed one (1YI6) is slightly different (29.0 and 23.9 ns respectively; values obtained by hydronmr program when considering residues 258-533). The phenomenon is likely be amplified in solution, where crystal constraints are not present and/or higher energy states can be accessed. For these reasons we decided to measure ^{15}N longitudinal and transversal relaxation rates (R1 and R2, respectively) and ^{1}H,^{15}N NOE in order to estimate the rotational tumbling time of the SrcP, when complexed with ATP.

Data are shown in Figure S9, compared with the theoretical values expected for the closed form of Src, based on its crystal structure. Values were calculated by hydronmr program using the pdb structure 1YI6, which is remarkably similar to the structure of the SrcP complexed with a nonhydrolyzable form of ATP (3DQX) but also contains the full activation loop. We observe a substantial agreement of R1 and R2 values, compatible with an overall tumbling of 20ns (23.9 estimated by hydronmr). This tumbling is lower than the one expected for free Src (29 ns based on pdb 2SRC) which was previously experimentally estimated 26 ns. This suggests a more compact conformation of the phosphorylated protein in the presence of the cofactor, in agreement with our model.

Relaxation experiments for the determination of the overall rotational correlation time for phosphorylated Src in the presence of ATP were performed at 700 MHz using pulse sequences for ^{15}N R_1 and R_2 rates, and heteronuclear NOE spectra (Figure S9) as described in the literature.

Relaxation delays for T1 were: 10, 200, 400, 600, 1000, 2000 ms while for T2 measurements we used the following delays: 8, 16, 32, 48, 80 and 128 ms.

The isotropic rotational correlation time τ_R (Figure S1) was estimated as:

$$\tau_R = \frac{1}{\omega_N}\sqrt{\frac{J(0) - J(\omega_N)}{J(\omega_N)}} \qquad (1)$$

where the spectral densities J are calculated from R_1,R_2 and NOE values as in [18].

In-gel digestion and LC-MS/MS analysis of peptides

Bands corresponding to phosphorylated and nonphosphorylated Src-KD were excised from the gel, washed, reduced with 15 mM TCEP in 50 mM ammonium bicarbonate for 45 min at 60°C and alkylated with 50 mM 2-chloroacetamide in 50 mM ammonium bicarbonate for 30 min at 25°C in the dark before digestion with proteomic grade trypsin (Promega, Madison, WI, USA) Extracted peptides were desalted using a C18 disk (Empore), eluted with 65% Acetonitrile, dried in a Speed-vacc and re-suspended in 0.1% formic acid in LC MS-grade water (Sigma Aldrich, St Louis, MI, USA). Peptides were separated by reversed-phase chromatography (Reprosil-Pur C18 3 µm, 200*0.075 mm, Dr. Maisch GmbH), using a nanoLCUltra1D+ system (Eksigent, Dublin CA, USA), directly coupled with a LTQ-OrbitrapVelos instrument (Thermo Fisher Scientific, Waltham MA, USA) via a nanoelectrospray source (Proxeon Biosystem).

MS spectra were acquired with an automatic switch between MS and MS/MS scans using a top five method with a threshold signal of 1000 counts. MS spectra were acquired with a resolution of 60000 (FWHM) at 400 m/z in the Orbitrap. Peptide fragmentation was performed with collision-induced dissociation (CID), and fragment ions were detected in the linear ion trap. The normalized collision energy was set to 35%, the Q-value was set to to 0.25, and the activation time was set to

10 msec. The maximum ion injection time for the MS/MS scans were 100 msec, and the ion target value was set to 5000.
Raw files were analyzed by the SequestHT search engine Proteome Discoverer (version 1.4.1.14) against a forward-reverse concatenated database (UniProtKB/Swiss-Prot, 88354 sequences, 26/03/2013 release). Oxidation of methionines was set as variable modification whereas carbamidomethylation of cysteines was considered as fixed modification in the SequestHT search engine. Minimal peptide length was set to 6 amino acids, a maximum of two missed-cleavages were allowed. Peptides were filtered at 1% FDR using Percolator.

Simulation details
The MD simulations were performed using GROMACS 4.6.7 with the PLUMED 2.1 plug-in for Metadynamics calculations. We used the Amber99SB*-ILDN force field[4] with the dihedral corrections of Best and Hummer[5]. The van der Waals interactions were cut-off at 1 nm and shifted to zero; the long-range electrostatic interactions were calculated by the particle mesh Ewald algorithm[6], with mesh spaced 0.12 nm. The system is coupled with a velocity-rescale thermostat[7] and the equations of motion are integrated with a time step of 2 fs. Each protein is enclosed in a dodecahedron box with periodic boundary conditions, the charges neutralized by an appropriate number of ions and solvated with tip3p water molecules[8]. Systems were equilibrated at constant pressure of 1 atm for 20 ns and subsequently at constant volume for 100 ns. Unbiased production simulations were run for 500 ns.

Enhanced sampling
PT-metaD[9] in the Well-Tempered Ensemble[10] has been performed for each system using 10 replicas at increasing temperatures (300.0, 314.0, 328.6, 343.9, 359.9, 376.6, 394.1, 412.4, 431.6 and 451.7 K) and attempting an exchange every 20 ps. All 10 replicas are subject to the well-tempered metadynamics[11] prescription in which a Gaussian is deposited in the collective variable (CV) space every 2 ps with height $W=W0*\exp[-V(s,t)/(f-1)T]$, where W0=10 kJ/mol is the initial height, T is the temperature of the replica, f=15 is the bias factor, and V(s,t) is the bias potential at time t and CV value s. The following three CVs are used: the antiparallel beta-sheet content between residues 381-384 and 408-411 (CV1), the alpha-helical content of residues 406-420 (CV2) and the center of mass distance between the Cα atoms of residues 408-418 and [381-384] U [437-440] (CV3). The widths of the Gaussian in the three CV dimensional space are s1=0.01, s2=0.03 and s3=0.01 nm. Note that CV1 and CV2 are dimensionless quantities. The specific choice of the CVs reflected previous experience[15-17] in which the importance of the beta-sheet and alpha-helix at the beginning of the A-loop were shown to be crucial for convergence. PTMetaD simulations were run for 1000 ns. Convergence was accessed monitoring the changes in the FES over time and the difference with the reconstruction obtain with the reweight algorithm of Tiwary and Parrinello[12].

Data analysis
The direction of the largest collective motion of the protein can be obtained doing a principal component analysis (PCA) on its trajectory. To this aim, we aligned the backbone atoms of the first 100 ns of the unphosphorylated Src trajectory at 300 K to the reference inactive structure and used the Cα atoms of the protein, excluding the first 10 residues and the activation loop residues, to calculate the covariance matrix. Projecting the trajectory on the first principal component vector (PC1) shows that the largest collective motion corresponds to a hinge motion of the N and C lobes. To relate this motion to the activation loop conformation, we calculated the Free Energy Surface (FES) as a function of PC1 and the center of mass distance of a short A-loop fragment (residues 411-413) to the αC-helix preceding turn (residues 301-303). This latter distance clearly distinguishes between an active-like and an inactive-like positioning of the A-loop. In order to project the FES on those variables, which are different from the biasing ones, we used the time-

independent reweighting approach by Tiwary et al.[12]. A script for the reweighting is freely available on the group website.

Supplementary Figures

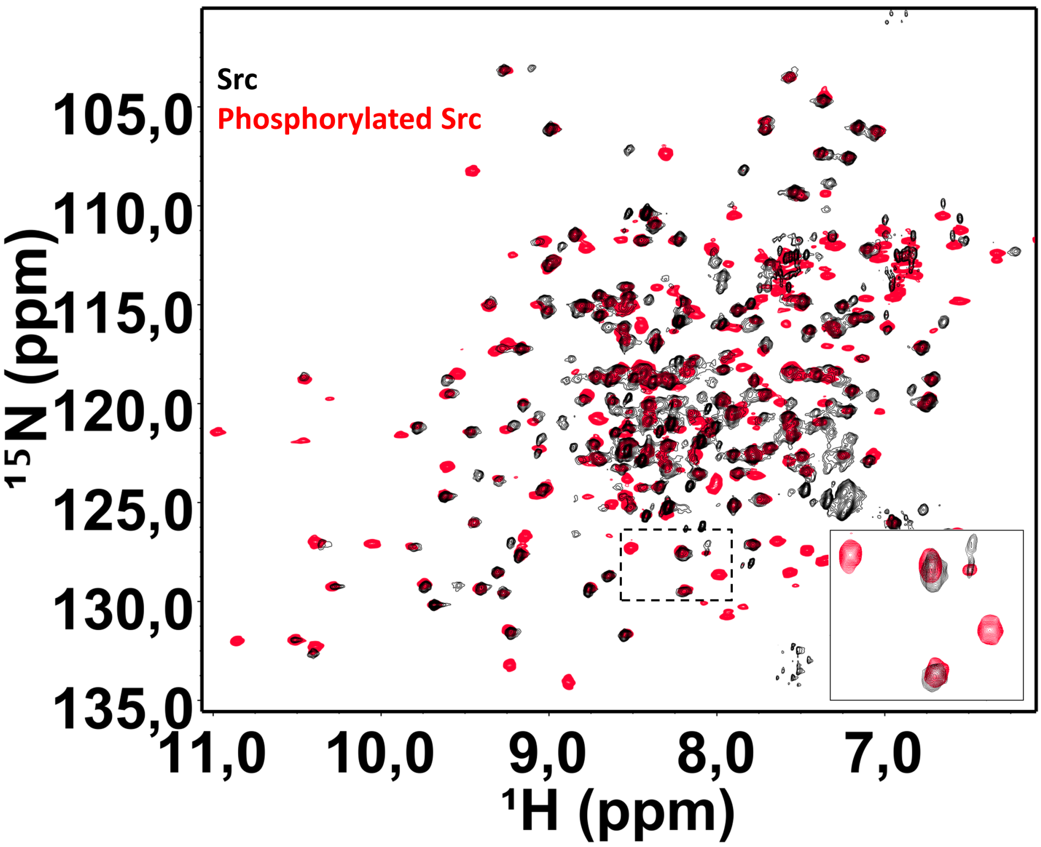

Figure S1. *^{1}H,^{15}N- TROSY NMR spectrum of the catalytic domain of Src (black) and its phosphorylated form (Y416, red). New peaks appear in the phosphorylated form (as better shown in the insert), few other disappear or shift.*

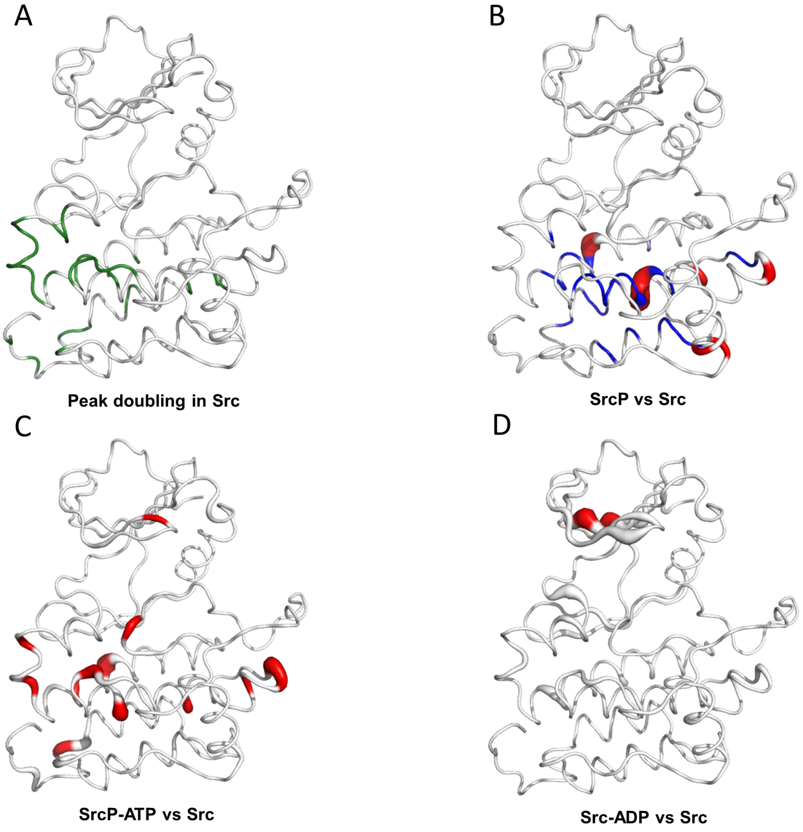

Figure S2. *Behavior of Src catalytic domain in different conditions as monitored by $^{1}H,^{15}N$- TROSY NMR spectrum. Doubling of peaks in free Src (A), effect of phosphorylation (B) and binding of cofactor on the phosphorylated (C) and unphosphorylated (D) protein are shown onto the phosphorylated crystallographyc structure (pdb code 1YI6). Peaks that appeared doubled are highlighted in green, peaks disappearing upon phosphorylation in blue,; backbone thickness is proportional to the combined amide chemical shift deviation; peaks shifted more than 0.1 ppm are colored in red.*

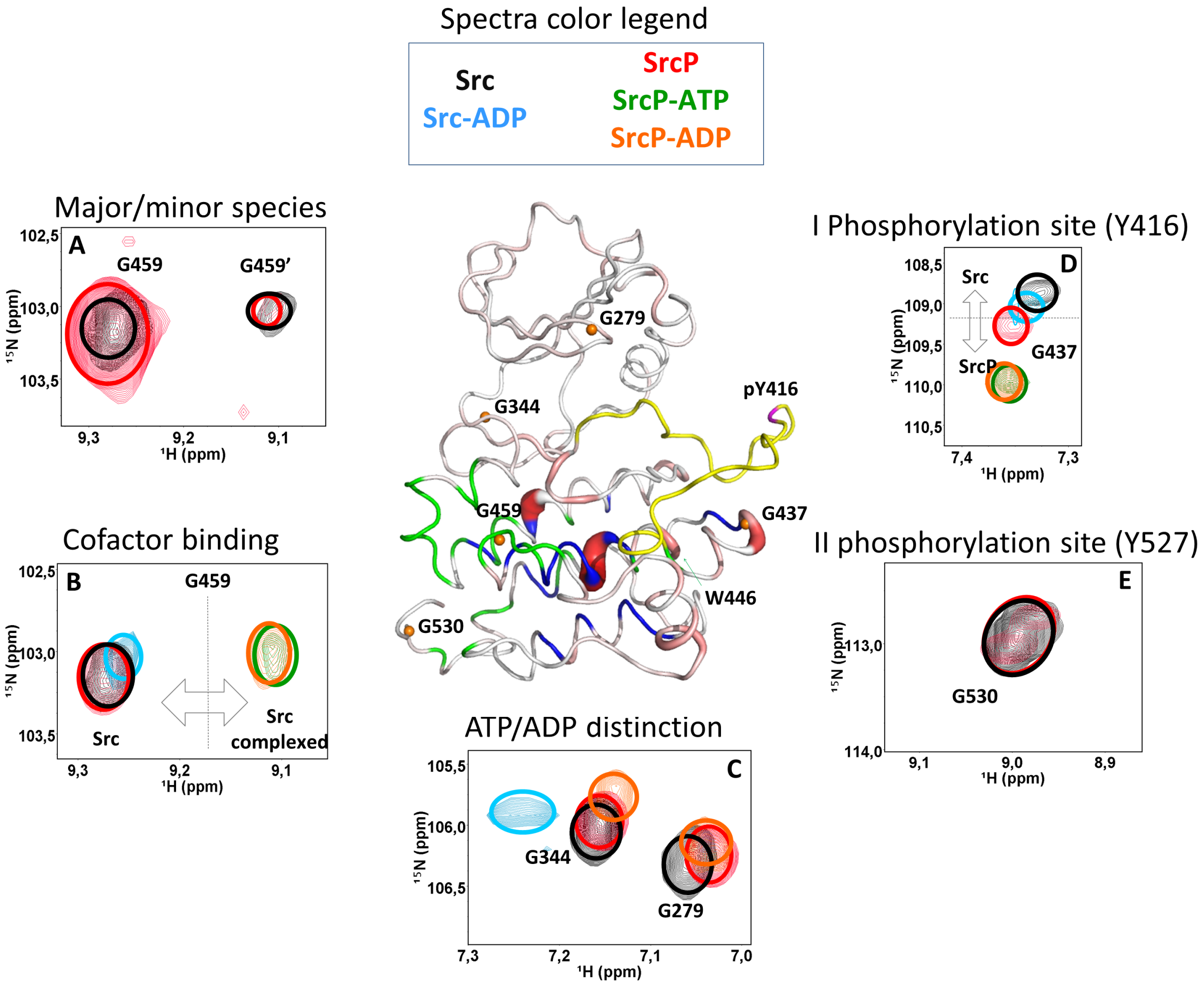

Figure S3. *The* $^{1}H,^{15}N$ *TROSY NMR glycine region provides a way to monitor the state of Src in a non-crowded region of the spectrum: (A) Src (black) and SrcP (red) exists in two unevenly populated forms in solution as exemplified by G459 (but also by many other peaks, see Fig 4); (B) the minor form (indicated by G459' in A) becomes prevalent with the addition of the cofactor (ATP/ADP, green and orange) only if the protein is phosphorylated. Addition of ADP to free Src (light blue) forms a weaker complex causing only a small shift. (C) Distinction between loading of ATP or ADP can be followed by monitoring G279 and G344, which shift or disappear in the presence of ADP (orange) or ATP (green), respectively. (D) Addition of ATP to Src phosphorylates only the first site while leaving the other unaltered (E). (Central) Crystal structure of Src (1YI6) indicating in green the residues for which the doubling of peaks is apparent in the spectrum. Peaks disappearing upon phosphorylation are also shown in blue. The selected residues of the panels are displayed as orange sphere in the structure, while the position of the phosphorylation site Y416 is in magenta. Colored circles have been added to better clarify the position of the peaks.*

Mass Spectrometry

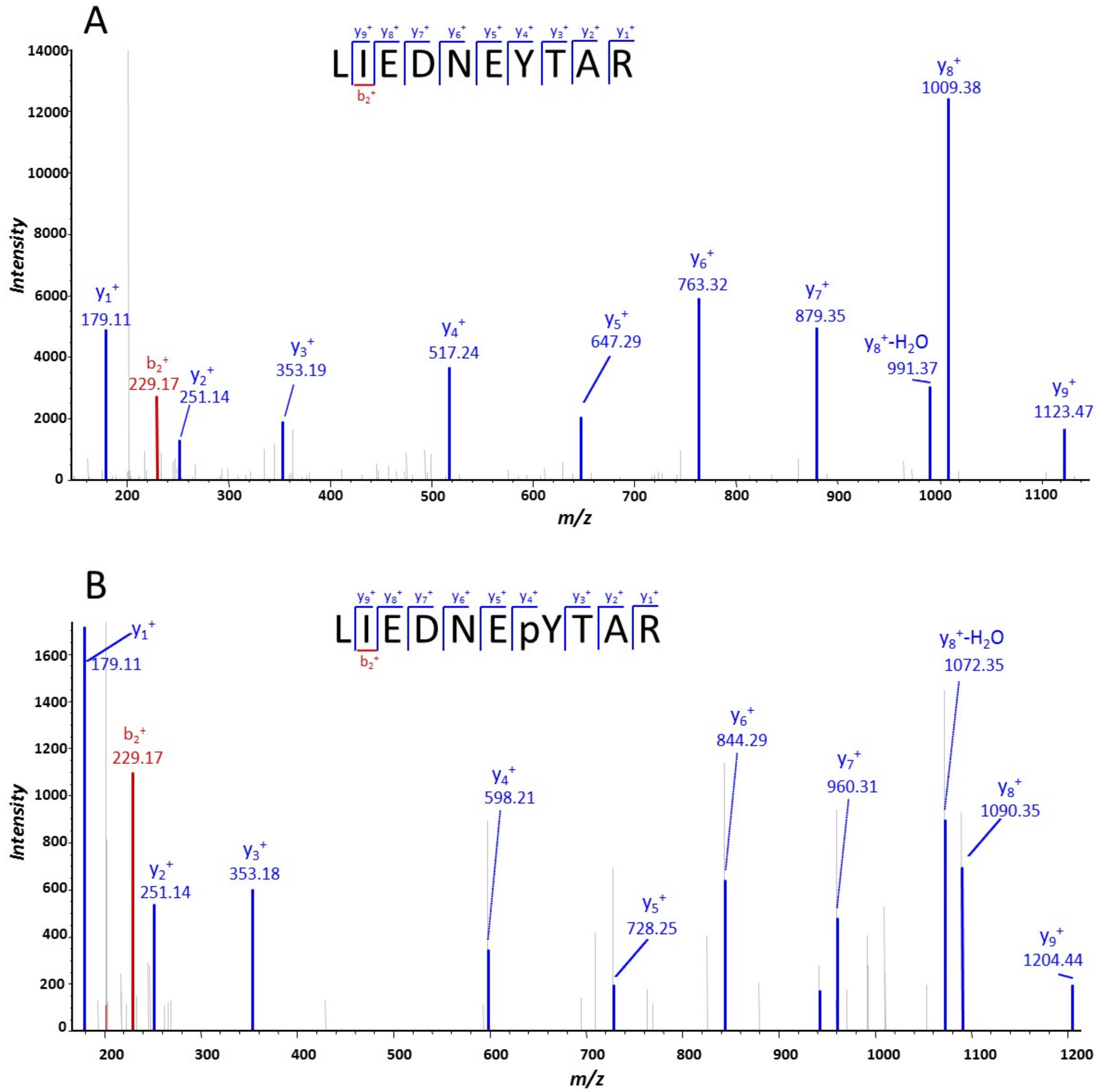

Figure S4. *Identification of phosphopeptides by mass spectrometry.* ***A)*** *MS/MS fragment spectrum corresponding to the tryptic non-phosphorylated Src 410-LIEDNEYTAR-419 peptide (m/z=619.27911 Da).* ***B)*** *MS/MS fragment spectrum of the tryptic phosphorylated Src 410-LIEDNEY*TAR-419 peptide (m/z=659.26200 Da) in which phosphorylation was unambiguously assigned to Y416 residue since the fragment ions y4-y9 showed corresponding neutral losses of phosphoric acid. In both panels, a good coverage of the whole sequence by 9y and 1b fragment ions is shown. Other b ions, although positively identified, are omitted for clarity.*

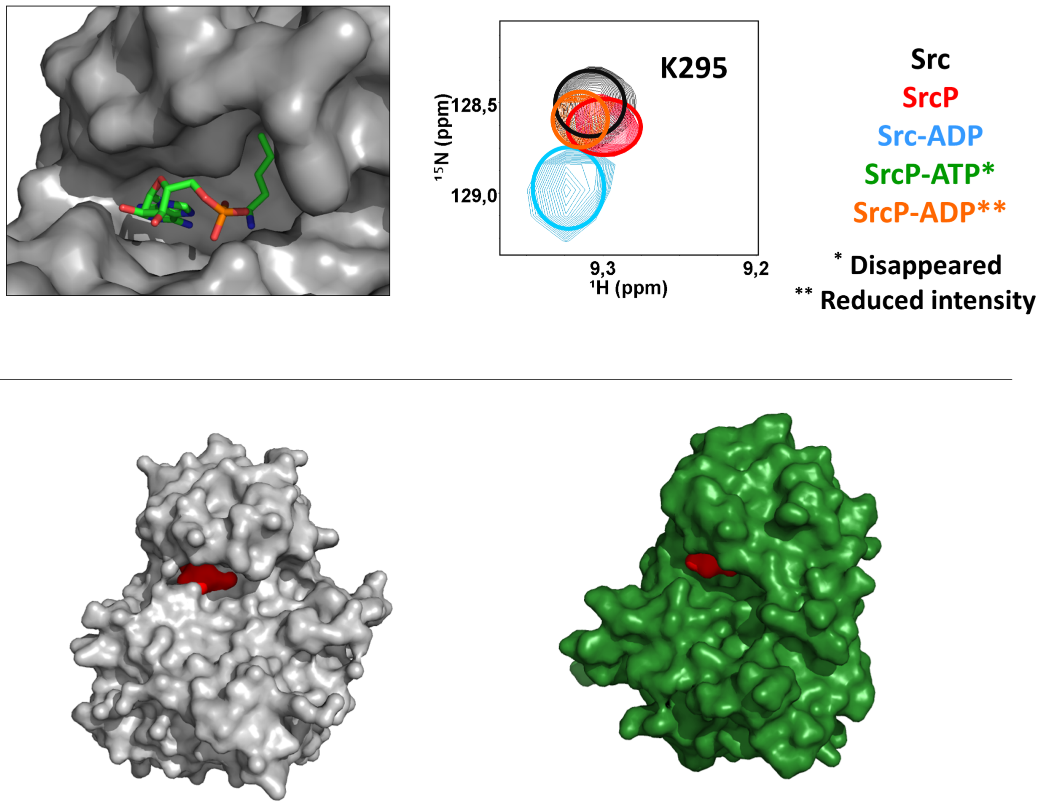

Figure S5. *(top left) the crystallographic structure of Src with AMP (pdb code 3DQX) . Positively charged K295 (depicted as sticks) is in close proximity to AMP. (top right) The amide signal of K295 in the ^{1}H,^{15}N TROSY spectrum disappears in the SrcP-ATP and SrcP-ADP complexes but not in the Src-ADP complex, in SrcP or in Src (respectively light blue, red and black peaks) supporting that phosphorylation is required for binding. (bottom) Comparison of the crystalloghraphic structures of phosphorylated Src (gray, pdb code 1YI6) and inactive unphosphorylated Src (green, pdb code 2SRC). The binding site (shown in red) is occluded by the activation loop in the unphosphorylated form explaining the weaker affinity of ADP for the unphosphorylated (vs phosphorylated) protein.*

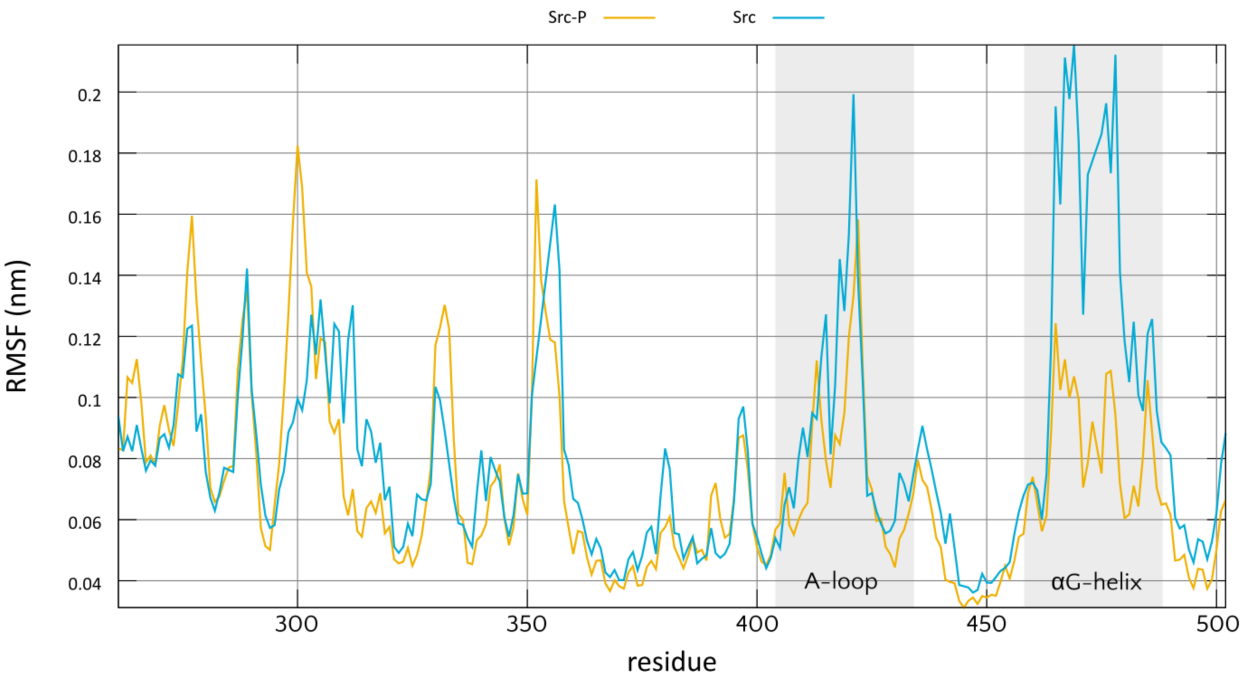

Figure S6. *Root mean square fluctuations (RMSF) of Src (blue) and Src phosphorilated (yellow). Lower fluctuations are observed on average for Src-P, in particular in important regions like the A-loop and the αG-helix.*

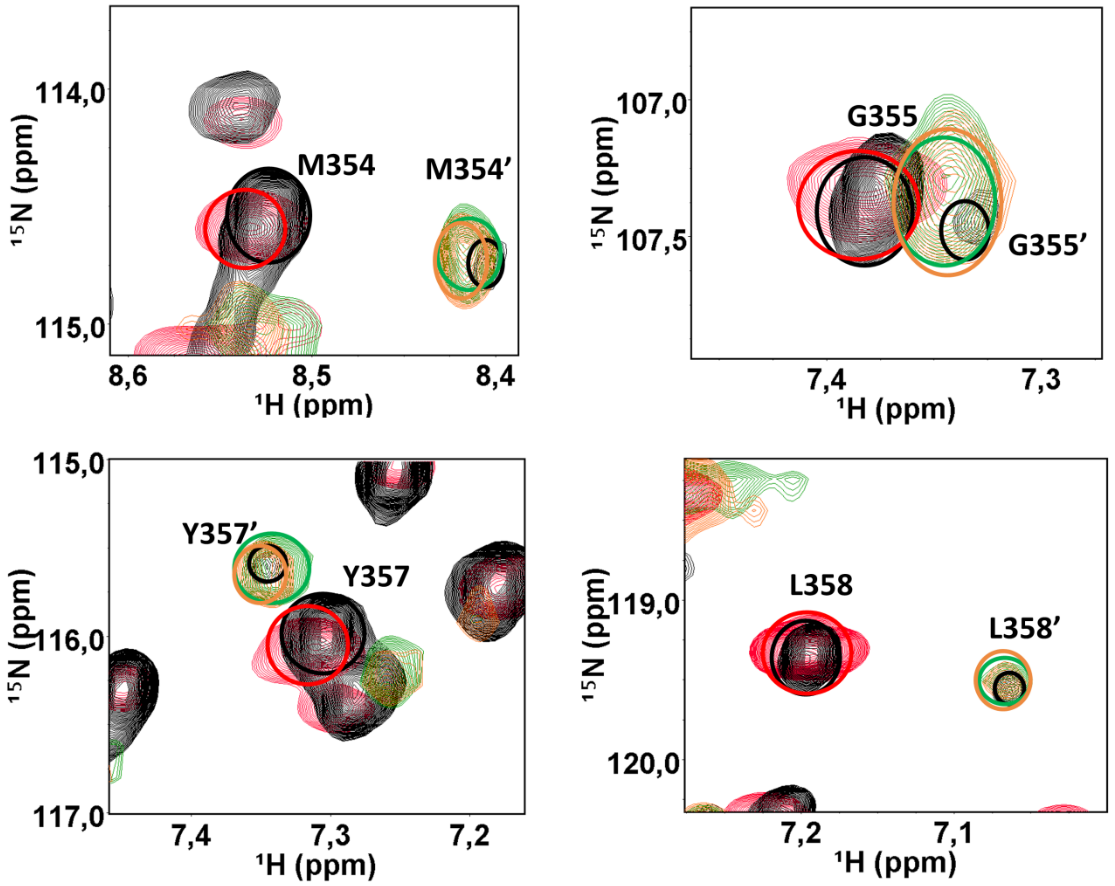

Figure S7. *A few examples from the $^{1}H,^{15}N$ TROSY NMR spectrum, of peak doubling in the unbound form of Src. Upon addition of the cofactor (ATP, green or ADP, orange), the position of the peaks in the complex becomes very similar to that found in the minor form (indicated by '). The effect is shown for residues 354, 355,357 and 358. In the panels the following color code is used: Src (black), SrcP (red), SrcP-ATP (green) and SrcP-ADP (orange). Colored circles have been added to better clarify the position of the peaks.*

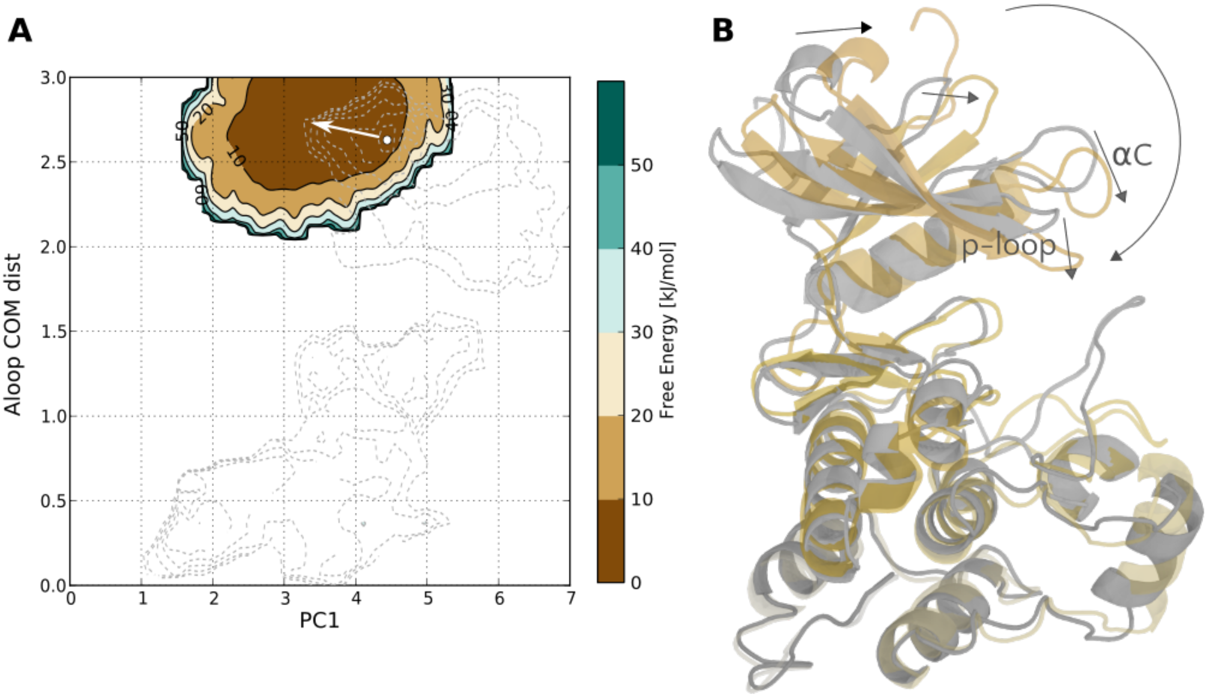

Figure S8. *(A) Free energy along the first PCA vector (PC1) and the A-loop distance (as in Figure 3) as obtained from a 700 ns unbiased MD simulations of Src-P bound to ATP. For reference, the free energy obtained for Src-P (as in Fig.3) is reported as dotted iso lines in grey. Starting from the main active minimum (white dot) the protein rapidly assumes a more compact structure, exploring lower values of the PC1 variables, corresponding to a smaller hinge opening. (B) Comparison of the initial (grey) and final (yellow) structure from the MD simulation with ATP. The closure of the hinge is evident, with the N-lobe closing onto the ATP site.*

Figure S9. 700 MHz ^{15}N R_1 (white bars), R_2 (yellow bars) relaxation rates (s-1) and ^{1}H,^{15}N NOE data (blue) measured for SrcP complexed with ATP. The correlation times (magenta bars) were calculated by equation (1). Theoretical values based on the crystal structure (1YI6) and calculated by hydronmr [2] are displayed as blue dots.

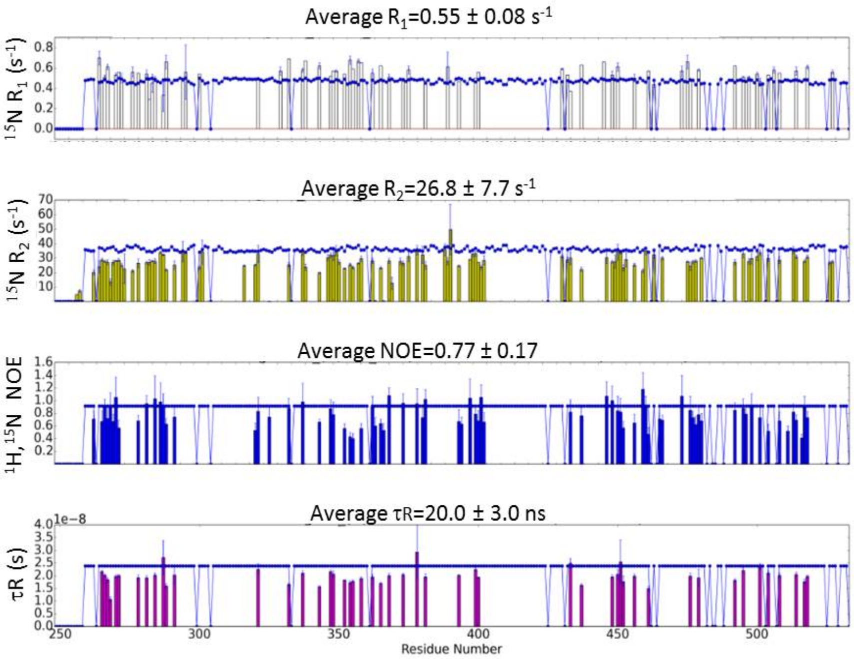

Table S1. Backbone ^{1}H, ^{15}N and ^{13}C NMR assignment of c-Src catalytic domain (residues 248-533) 0.2 mM in phosphate buffer 20 mM at pH 6.4, NaCl 0.5 M, TCEP 1mM, NaN3 0.03%, MgCl2 1mM, T=293K. The table also indicates peaks which appear to be doubled (existing in a minor and a major form) and those which disappear or weaken upon protein phosphorylation.

Residue	res type	HN	N	Cα	Cβ	C'	Doubled (dd)	Disappear/weaken in SrcP (d)
248	gly	-	-	-	-	-		
249	his	-	-	-	-	-		
250	met	-	-	-	-	-		
251	gln	8.53	122.4	56.5	28.8	176.3		
252	thr	8.22	115.7	62.2	69.6	174.6		
253	gln	8.44	122.8	56.2	29.3	176.4		
254	gly	8.42	110.3	45.2	-	174.0		
255	leu	8.10	121.8	55.1	42.5	177.2		
256	ala	8.29	125.2	52.5	19.3	177.7		
257	lys	8.27	120.9	56.4	32.9	176.3		
258	asp	8.42	122.2	53.8	42.0	-		
259	ala	-	-	-	-	177.8		
260	trp	8.43	118.3	58.2	29.2	-		
261	glu	-	-	56.9	-	177.7		
262	ile	8.16	120.4	58.9	39.8	-		
263	pro	-	-	62.0	-	179.6		
264	arg	9.40	129.4	60.0	29.6	178.0		
265	glu	9.36	114.9	58.5	27.8	176.9		
266	ser	7.87	115.3	59.9	63.8	173.2		
267	leu	7.88	123.4	53.3	45.4	175.9		
268	arg	8.52	121.3	54.8	32.3	174.8		
269	leu	8.77	129.5	57.3	39.4	176.3		
270	glu	8.73	122.8	58.9	31.5	176.3		
271	val	8.14	117.9	61.4	36.5	173.3		
272	lys	8.55	131.8	57.5	30.5	175.8		
273	leu	9.27	129.7	55.4	42.5	177.8		
274	gly	7.69	105.8	45.3	-	171.0		
275	gln	8.37	116.9	55.3	31.3	174.4		
276	gly	8.38	111.1	44.4	-	-		
277	cys	-	-	60.3	32.1	176.7		
278	phe	8.39	118.8	51.0	40.5	174.7		
279	gly	7.05	106.3	45.4	-	172.5		
280	glu	8.31	118.7	55.4	33.8	175.2		
281	val	8.25	119.9	62.0	33.1	174.9		
282	trp	9.80	127.3	56.5	31.8	175.5		
283	met	9.79	121.2	54.9	34.5	176.5		
284	gly	9.01	115.4	46.0	-	172.3		
285	thr	8.63	114.8	61.2	71.7	173.4		
286	trp	9.75	129.4	53.5	32.3	175.4		
287	asn	9.63	124.8	54.3	35.9	175.5		
288	gly	8.99	106.0	46.1	-	175.2		
289	thr	7.54	109.2	62.4	71.1	174.6		
290	thr	7.92	120.4	63.0	70.4	173.3		
291	arg	9.23	131.6	57.0	29.3	175.3		
292	val	8.55	116.8	60.2	35.9	174.0		
293	ala	8.69	122.8	50.7	20.4	176.4		
294	ile	9.47	121.4	60.8	41.4	174.6		
295	lys	9.31	128.5	55.6	33.7	-		
296	thr	-	-	-	-	-		
297	leu	-	-	-	-	-		
298	lys	-	-	-	-	-		
299	pro	-	-	64.3	31.4	177.5		
300	gly	9.08	111.9	45.3	-	-		
301	thr	7.99	112.4	-	-	-		
302	met	-	-	-	-	-		
303	ser	-	-	-	-	-		
304	pro	-	-	66.2	-	178.0		
305	glu	8.57	115.3	60.1	28.5	179.0		
306	ala	7.78	122.7	54.8	18.1	-		
307	phe	-	-	-	-	-		
308	leu	-	-	-	-	-		
309	gln	-	-	-	-	-		
310	glu	-	-	-	-	-		
311	ala	-	-	-	-	-		

312	gln	-	-	-	-	-		
313	val	-	-	-	-	-		
314	met	-	-	-	-	-		
315	lys	-	-	-	-	177.6		
316	lys	7.11	115.6	56.1	32.4	-		
317	leu	-	-	-	-	-		
318	arg	-	-	-	-	-		
319	his	-	-	57.6	33.1	174.9		
320	glu	7.74	124.8	59.3	29.5	176.6		
321	lys	10.46	118.5	53.3	29.9	174.6		
322	leu	7.92	120.8	53.7	43.2	-		
323	val	-	-	-	-	-		
324	gln	-	-	54.8	-	175.6		
325	leu	8.52	125.3	56.0	42.5	175.8		
326	tyr	9.31	123.9	55.2	39.0	-		
327	ala	-	-	-	-	-		
328	val	-	-	60.1	36.4	173.7		
329	val	8.53	121.9	-	-	-		
330	ser	-	-	60.1	62.3	173.5		
331	glu	6.72	119.8	54.2	31.4	175.1		
332	glu	8.57	122.5	-	-	-		
333	pro	-	-	-	-	-		
334	ile	-	-	-	-	-		
335	tyr	-	-	52.6	42.5	176.1		
336	ile	9.16	120.0	62.5	-	174.7		
337	val	8.64	128.7	61.6	-	175.6		
338	thr	9.59	119.5	59.8	73.4	-		
339	glu	-	-	-	-	-		
340	tyr	-	-	-	-	-		
341	met	-	-	-	-	-		
342	ser	-	-	-	-	176.0		
343	lys	8.40	120.0	55.2	31.1	177.0		
344	gly	7.16	106.0	45.0	-	172.5		
345	ser	8.80	115.1	57.8	64.6	176.3		
346	leu	9.45	126.0	57.4	40.6	177.4		
347	leu	6.78	117.2	58.1	40.9	176.8		
348	asp	7.18	115.8	57.0	39.7	179.0		
349	phe	8.56	123.3	60.8	39.1	178.3	dd	
350	leu	8.51	116.8	57.5	42.0	176.8	dd	d
351	lys	7.09	112.1	57.3	33.2	177.8	dd	
352	gly	7.22	107.6	44.5	-	175.1		
353	glu	8.64	118.8	59.5	29.4	178.4	dd	
354	met	8.53	114.6	56.7	30.8	179.2	dd	
355	gly	7.38	107.4	46.8	-	176.4	dd	
356	lys	7.40	118.5	58.2	31.4	177.5	dd	
357	tyr	7.31	116.0	58.0	38.1	176.3	dd	
358	leu	7.19	119.4	55.8	42.8	176.9	dd	
359	arg	8.61	121.5	53.2	32.7	-	dd	
360	leu	-	-	-	-	-		
361	pro	-	-	66.8	29.9	179.7		
362	gln	7.25	115.1	59.6	29.0	178.0	dd	
363	leu	7.80	119.6	58.3	41.5	179.2		d
364	val	8.90	118.9	67.0	31.4	177.0	dd	d
365	asp	7.58	120.8	58.0	41.5	179.1	dd	
366	met	8.22	118.7	60.9	-	-	dd	d
367	ala	-	-	55.6	-	179.0		
368	ala	9.07	121.9	56.7	17.2	180.7		d
369	gln	8.34	119.9	60.3	-	-		
370	ile	-	-	61.6	-	178.2		
371	ala	8.25	119.6	-	-	178.0	dd	
372	ser	8.01	113.0	60.6	-	178.4		
373	gly	7.84	108.2	47.5	-	-		
374	met	-	-	53.8	35.8	178.3		
375	ala	8.86	125.0	55.0	-	180.6		d
376	tyr	7.26	122.7	61.6	36.6	-		
377	val	-	-	67.8	30.9	177.4		
378	glu	8.83	118.8	59.4	31.3	-		
379	arg	-	-	-	-	177.6		
380	met	7.71	116.9	54.0	30.3	175.9		
381	asn	7.96	113.5	55.1	37.1	-		
382	tyr	-	-	-	-	-		
383	val	-	-	-	-	-		
384	his	-	-	-	-	-		

385	arg	-	-	-	-	-		
386	asp	-	-	-	-	-		
387	leu	-	-	56.4	41.1	173.6		
388	arg	6.65	115.9	54.6	30.2	176.5		
389	ala	10.36	127.2	56.5	-	178.8		
390	ala	9.61	118.9	54.5	-	-		
391	asn	-	-	58.7	-	176.9		
392	ile	7.34	118.3	56.8	-	175.4		
393	leu	8.82	126.8	53.0	-	174.6		
394	val	8.52	120.4	60.6	32.8	175.3		
395	gly	8.82	115.1	43.1	-	173.4		
396	glu	8.28	118.8	56.8	29.1	177.9		
397	asn	9.17	117.2	54.7	37.1	174.2		
398	leu	8.46	111.7	56.0	37.9	175.6		
399	val	6.72	118.8	62.6	32.1	175.2		
400	cys	8.12	123.6	57.6	31.0	172.7		
401	lys	8.54	114.1	54.2	38.4	175.3		
402	val	8.82	122.7	-	31.0	-		
403	ala	-	-	-	-	-		
404	asp	-	-	-	-	-		
405	phe	-	-	-	-	-		
406	gly	-	-	-	-	-		
407	leu	-	-	-	-	-		
408	ala	-	-	-	-	-		
409	arg	-	-	-	-	-		
410	leu	-	-	-	-	-		
411	ile	-	-	-	-	-		
412	glu	-	-	-	-	-		
413	asp	-	-	-	-	-		
414	asn	-	-	-	-	-		
415	glu	-	-	-	-	-		
416	tyr	-	-	-	-	-		
417	thr	-	-	-	-	-		
418	ala	-	-	-	-	-		
419	arg	-	-	-	-	-		
420	gln	-	-	-	-	-		
421	gly	-	-	-	-	-		
422	ala	-	-	-	-	-		
423	lys	-	-	-	-	-		
424	phe	-	-	-	-	-		
425	pro	-	-	-	-	-		
426	ile	-	-	-	-	-		
427	lys	-	-	-	-	-		
428	trp	-	-	-	-	-		
429	thr	-	-	-	-	177.9		
430	ala	8.94	120.9	-	-	-	dd	
431	pro	-	-	65.4	30.3	176.9		
432	glu	9.04	114.8	58.7	25.5	177.7		
433	ala	6.63	126.6	54.1	-	177.0		
434	ala	7.57	120.1	54.7	-	-		
435	leu	-	-	-	-	178.8		
436	tyr	6.22	112.3	-	-	176.6		
437	gly	7.34	108.8	46.3	-	174.1		
438	arg	7.19	121.8	55.0	28.3	174.3		d
439	phe	8.20	125.3	57.6	39.0	-		d
440	thr	-	-	-	-	-		
441	ile	-	-	-	-	-		
442	lys	-	-	55.8	-	182.2		
443	ser	8.61	120.4	61.9	-	-		
444	asp	-	-	58.7	39.6	179.4		
445	val	8.27	123.3	67.4	30.7	178.0		
446	trp	7.45	121.3	61.6	27.3	179.7	dd	d
447	ser	8.64	116.2	62.1	63.8	176.6		d
448	phe	9.41	123.7	62.0	38.6	176.9		
449	gly	8.53	107.2	47.4	-	176.0		
450	ile	8.10	119.7	63.0	-	180.5		d
451	leu	9.54	129.3	59.0	-	179.8		d
452	leu	8.51	119.1	58.6	-	180.6		d
453	thr	7.97	114.1	66.2	68.3	176.2		d
454	glu	7.75	125.0	61.1	31.0	178.7	dd	d
455	leu	8.37	116.5	58.8	42.2	177.9		d
456	thr	7.56	103.4	64.5	70.2	175.4		
457	thr	7.48	109.3	61.7	69.3	174.3		

458	lys	7.95	118.3	58.4	28.8	175.2	dd	
459	gly	9.27	103.2	45.3	-	173.6	dd	
460	arg	7.49	122.7	56.6	29.5	176.5	dd	
461	val	8.20	127.6	60.7	32.3	-	dd	
462	pro	-	-	62.3	32.4	174.4		
463	tyr	7.49	114.8	57.4	36.6	-		
464	pro	-	-	64.1	30.2	178.8		
465	gly	8.84	111.4	45.0	-	173.8		
466	met	7.97	119.8	55.2	34.9	176.6		
467	val	8.59	118.3	60.6	32.5	-		
468	asn	-	-	57.1	-	177.5		
469	arg	8.55	116.1	58.7	29.1	-		
470	glu	-	-	57.3	31.5	177.4		
471	val	7.28	118.8	66.8	31.0	-		
472	leu	-	-	58.2	40.9	178.6		
473	asp	7.95	117.6	57.5	40.7	178.8		
474	gln	7.80	116.1	59.1	26.1	179.6		
475	val	9.07	120.7	66.2	-	181.7		
476	glu	8.60	124.3	59.8	-	177.8		
477	arg	7.27	116.2	57.0	30.2	176.6		
478	gly	7.71	106.1	45.0	-	174.2		
479	tyr	8.34	124.2	60.8	38.6	175.8		
480	arg	6.94	126.0	52.3	32.7	174.3		
481	met	7.72	119.5	55.7	33.4	-		
482	pro	-	-	-	-	-		
483	cys	-	-	-	-	-		
484	pro	-	-	-	-	-		
485	pro	-	-	64.4	31.5	177.8		
486	glu	8.70	115.5	58.6	28.1	174.3	dd	
487	cys	7.91	122.6	56.0	28.7	-		
488	pro	-	-	-	-	-		
489	glu	-	-	-	-	-		
490	ser	-	-	60.6	62.0	177.7		
491	leu	7.47	123.5	56.6	41.7	178.4		
492	his	7.57	121.6	59.5	30.6	177.6		d
493	asp	9.20	121.1	57.4	40.1	178.3		
494	leu	6.75	120.0	56.5	41.2	179.3		
495	met	7.46	118.6	60.0	29.9	175.9		d
496	cys	7.53	111.9	63.6	26.0	178.4		
497	gln	7.54	121.2	59.6	27.6	178.3		
498	cys	6.99	116.1	62.9	25.2	173.7		d
499	trp	6.52	111.8	52.0	26.5	176.2		d
500	arg	6.84	119.2	56.9	29.1	177.7		d
501	lys	9.03	124.5	60.6	32.3	-		
502	asp	-	-	-	-	-		
503	pro	-	-	65.0	32.7	178.6		
504	glu	8.22	111.7	58.1	28.5	178.1		
505	glu	7.77	115.8	56.1	30.2	177.0		
506	arg	7.36	121.0	54.9	29.3	-		
507	pro	-	-	62.2	32.6	173.9		
508	thr	7.37	104.7	59.5	70.3	-		
509	phe	-	-	63.8	-	178.7		
510	glu	8.42	121.0	60.3	29.6	178.8		
511	tyr	8.30	122.1	61.3	37.2	177.6		
512	leu	8.43	120.7	58.3	-	177.9		
513	gln	8.87	117.8	60.5	26.8	177.0	dd	d
514	ala	7.22	120.3	55.2	17.7	179.6	dd	
515	phe	8.61	118.7	60.4	38.7	179.0		d
516	leu	8.27	118.0	57.6	-	179.1		d
517	glu	8.62	119.9	59.8	29.1	178.5		
518	asp	7.45	116.3	54.1	40.6	177.2		
519	tyr	7.09	122.9	63.0	39.6	176.8	dd	
520	phe	8.70	114.5	60.5	37.4	174.5	dd	
521	thr	7.69	112.8	63.8	68.8	175.5		
522	ser	8.20	114.8	60.0	63.2	-		
523	thr	-	-	-	-	-		
524	glu	-	-	-	-	-		
525	pro	-	-	64.1	31.5	177.8		
526	gln	8.49	118.6	55.2	28.3	175.5		
527	tyr	7.68	122.2	59.8	38.4	175.0	dd	
528	gln	7.79	127.3	52.4	30.2	-		
529	pro	-	-	60.5	32.6	178.6		
530	gly	9.00	113.0	44.3	-	-		

531	glu	-	-	-	-	-		
532	asn	-	-	54.2	40.6	173.7		
533	leu	7.90	125.0	56.2	45.2	-		

Table S2. Temptative backbone ^{1}H, ^{15}N and ^{13}C NMR assignment of the activation loop (and parts of αC helix) for phosphorylated c-Src catalytic domain 0.3 mM in phosphate buffer 20 mM at pH 6.4, NaCl 0.5 M, TCEP 1mM, NaN3 0.03%, MgCl2 1mM, T=293K. Assignment is based on both inter-residues connectivity and SPARTA+ chemical shift prediction (see experimental part), in the assumption that new spin systems detected upon Src phosphorylation, arise from the activation loop or the αC helix.

Res	HN	N	$C\alpha$	$C\beta$	$C\alpha_{-1}$	$C\beta_{-1}$	C'_{-1}	Spin Type (res-1)res	Candidates sorted by SPARTA+
**303**	8.77	119.7	57.2	63.2	55.4	35.2	174.1	(CKM)S	S303
**308**	8.14	115.4	56.9	40.1	61.4	-	175.2	(CIFPSTYV)NDLFY	L308
**311**	7.93	130.8	50.9	23.3	-	-	173.8	(all)A	A422,A311,A408,A418
**313**	11.86	132.2	62.7	34.8	59.8	-	173.6	(RCQEHIKFSTWYV)V	V313
**405**	8.35	114.2	56	40.7	54.9	-	179.2	(ARDQELKMW)NDLFY	F405,L410,N414,Y416,F424,D413
**406**	9.45	108.3	44.6	-	-	-	170.5	(all)G	G421,G406
**407**	10.05	127.2	52.5	42.5	-	-	175	(all)DL	D413,L410,L407,L317,D404,L308
**408**	8.33	122.7	53.9	18.6	-	42.5	177.1	(DGLFY)A	A408,A422
**409**	7.21	117.3	58.6	-	-	-	177.6	(all)RCQEHILKMFSWY	L308,R409,E310,K427,K315,R419,M314,I411,E412,K423,W428,I426,L317,L407,Q309,Q420,E415,Y416,F307,Q312,F405,S303,F424,L410
**410**	8.62	125.2	57.1	39.5	-	31.1	174.4	(RCQEGHKMPWV)DLFY	L410,D413,F424,Y416,L407
**411**	7.94	117.9	58.6	40.8	-	-	176.8	(all)ILFY	L317,L407,L308,Y416,F405,I411,F424,I426,L410,F307
**412**	7.34	117.2	59.2	32.9	-	-	176.8	(all)RCEHKMWV	E412,E415,R409,E310,K427,K315,R419,M314,V313,K423,W428
**413**	9.06	124.7	56.8	41.4	-	-	175.8	(all)NDLFY	Y416,F405,D413,L407,L317,F424,N414,D404,L308,L410,F307
**414**	8.51	115.1	55.9	-	-	-	177.2	(all)ARNDCQEHLKMFSWY	N414,D404,L308,E412,R409,E310,K427,L317,K315,L407,R419,M314,Q420,K423,A408,A311,E415,W428,Y416,F405,Q309,D413,A422,S303,F424,F307,Q312,L410,A418
**416**	8.29	125.7	57.5	-	60.7	-	175.7	(CEIFPSTWYV)RDCQEHLKMFSWY	L308,E412,K427,M314,D413,Y416
**417**	7.5	118.7	63.5	70.3	-	-	175.1	(all)T	T417, T429
**418**	8.13	117.7	52.6	23.2	-	-	176	(all)A	A422,A311,A408,A418
**419**	8.77	112.2	58.2	-	-	-	177	(A)RCQEHILKMFSWY	R419,R409,Q312,K423
**421**	8.56	111.7	46*	-	-	-	175.9	all(G)	G421,G406

**422**	7.77	123.7	53.2	19.5	46	-	174.2	(G)A	A422
**423**	8.12	120.9	56	33.1	53.2	19.5	177	(A)RCEHKMW	K423,R409,R419
**426**	7.63	127.1	58.9	40	64.3	-	178.3	(IPV)ILFY	I426,
**427**	7.86	120.5	55.4	35.1	-	-	174.9	(all)CKM	K427,K315,M314, K423
**428**	9.24	117.1	61.1	31.6	-	-	177.4	(all)CWV	V313, W428
**429**	8.25	116.4	62.4	70	-	-	-	(all)T	T417,T429
*peak almost lost in the noise									

References

[1] T. Takeya, H. Hanafusa, *Cell* **1983**, *32*, 881–890.
[2] J. B. Bliska, K. L. Guan, J. E. Dixon, **1991**.
[3] R. Campos-Olivas, M. Marenchino, L. Scapozza, F. L. Gervasio, *Biomol Nmr Assigm* **2011**, *5*, 221–224.
[4] K. Lindorff-Larsen, S. Piana, K. Palmo, P. Maragakis, J. L. Klepeis, R. O. Dror, D. E. Shaw, *Proteins* **2010**, *78*, 1950–1958.
[5] R. B. Best, G. Hummer, *J Phys Chem B* **2009**, *113*, 9004–9015.
[6] U. Essmann, L. Perera, M. Berkowitz, T. Darden, H. Lee, L. Pedersen, *J Chem Phys* **1995**, *103*, 8577–8593.
[7] G. Bussi, D. Donadio, M. Parrinello, *J Chem Phys* **2007**, *126*, 014101.
[8] W. Jorgensen, J. Chandrasekhar, J. Madura, R. Impey, M. Klein, *J Chem Phys* **1983**, *79*, 926–935.
[9] G. Bussi, F. Gervasio, A. Laio, M. Parrinello, *J Am Chem Soc* **2006**, *128*, 13435–13441.
[10] M. Bonomi, M. Parrinello, *Phys Rev Lett* **2010**, *104*, 190601.
[11] A. Barducci, G. Bussi, M. Parrinello, *Phys Rev Lett* **2008**, *100*, 020603.
[12] P. Tiwary, M. Parrinello, *J Phys Chem B* **2015**, *119*, 736–742.
[13] Y. Shen, A. Bax , *J Biomol NMR* **2010,** 48:13–22
[14] F.H. Schumann , H. Riepl , T. Maurer , W. Gronwald , K.P. Neidig , H.R. Kalbitze , *J Biol NMR* **2007,** 39:275-289
[15] Lovera, S., Morando, M., Pucheta-Martinez, E., Martinez-Torrecuadrada, J. L., Saladino, G., & Gervasio, F. L. *PLoS Comput Biol*, **2015**, *11*(11), e1004578.
[16] Lovera, S., Sutto, L., Boubeva, R., Scapozza, L., Dölker, N., & Gervasio, F. L. *Journal of the American Chemical Society*, **2012,** *134*(5), 2496–9.
[17] Sutto, L., & Gervasio, F. L.. *Proc Nat Acad Sci USA* **2013,** *110*(26), 10616–21.
[18] Bentrop, D. et al. Structural and dynamical properties of a partially unfolded Fe4S4 protein: role of the cofactor in protein folding. *Biochemistry* **1999**, 38, 4669–4680.
